# Mastication and Risk for Diabetes in a Japanese Population: A Cross-Sectional Study

**DOI:** 10.1371/journal.pone.0064113

**Published:** 2013-06-05

**Authors:** Toru Yamazaki, Masashi Yamori, Keita Asai, Ikuko Nakano-Araki, Akihiko Yamaguchi, Katsu Takahashi, Akihiro Sekine, Fumihiko Matsuda, Shinji Kosugi, Takeo Nakayama, Nobuya Inagaki, Kazuhisa Bessho, for the Nagahama Study Collaboration Group

**Affiliations:** 1 Department of Oral and Maxillofacial Surgery, Graduate School of Medicine, Kyoto University, Kyoto, Japan; 2 Department of Oral and Maxillofacial Surgery, Nagahama City Hospital, Nagahama, Japan; 3 EBM Research Center, Graduate School of Medicine, Kyoto University, Kyoto, Japan; 4 Unit of Human Disease Genomics, Center for Genomic Medicine, Graduate School of Medicine, Kyoto University, Kyoto, Japan; 5 Department of Medical Ethics, School of Public Health, Graduate School of Medicine, Kyoto University, Kyoto, Japan; 6 Department of Health Informatics, School of Public Health, Graduate School of Medicine, Kyoto University, Kyoto, Japan; 7 Department of Diabetes and Clinical Nutrition, Graduate School of Medicine, Kyoto University, Kyoto, Japan; McGill University, Canada

## Abstract

**Background:**

Associations between mastication and insufficient nutrient intake, obesity, and glucose metabolism have been shown in previous studies. However, the association between mastication and diabetes has not been clarified. Our objective was to examine the association between mastication, namely masticatory performance or rate of eating, and diabetes in a population-based cohort.

**Methods:**

We conducted a cross-sectional study of the association between mastication and diabetes in the Nagahama Prospective Cohort Study, an ongoing study which recruits citizens of Nagahama City in Shiga Prefecture, central Japan. 2,283 male and 4,544 female residents aged 40–74 years were enrolled from July 2009 to November 2010. Masticatory performance was evaluated by spectrophotometric measurement of color changes after masticating color-changeable chewing gum. Categorical rate of eating (fast, intermediate or slow) was self-assessed using a questionnaire.

**Results:**

177 males (7.7%) and 112 (2.4%) females were diagnosed with diabetes. We divided participants into four groups by quartile of masticatory performance, namely Q1 (lowest), 2, and 3 and 4 (highest). Compared to the lowest performance group, the multivariable adjusted odds ratio (OR) of diabetes was 0.91 (95% confidence interval (CI), 0.58–1.4) in Q2, 0.77 (95% CI, 0.48–1.2) in Q3, and 0.53 (95% CI, 0.31–0.90) in the highest group in males, and 1.2 (95% CI, 0.73–2.0), 0.95 (95% CI, 0.54–1.6) and 0.56 (95% CI, 0.30–1.0) in females. We also estimated ORs of diabetes by rate of eating. Compared to the fast eating group, ORs in males were 0.87 (95% CI, 0.61–1.2) in the intermediate group and 0.38 (95% CI, 0.16–0.91) in the slow group, and ORs in females were 0.92 (95% CI, 0.59–1.4) and 1.5 (95% CI, 0.73–3.0).

**Conclusions:**

These findings support the hypothesis that higher masticatory performance and slow eating prevent the occurrence of diabetes.

## Introduction

Diabetes is a group of metabolic diseases which is characterized by hyperglycemia resulting from defects in insulin secretion, insulin resistance, or both. The pathogenesis of diabetes involves lifestyles and environmental factors [1], which include physical inactivity, insufficient intake of nutrients, or obesity, which often arises due to excessive intake of food or fast eating [2]–[7]. Several studies have shown that lifestyle and diet modifications may play an important role in its prevention [8], [9].

A number of studies have identified associations between mastication and insufficient nutrient intake. People who were unable to fully masticate due to teeth loss or ill-fitting dentures had insufficient daily nutrient intake of dietary fiber, magnesium or calcium [10]–[12], nutrients which may be protective against type 2 diabetes [3], [4]. These results suggest that higher masticatory performance may contribute to a lower risk of diabetes.

On the other hand, a few studies have demonstrated direct relationships between mastication and glucose metabolism. Thorough mastication elicited a lower postprandial plasma glucose concentration because of the potentiation of early-phase insulin secretion [13]. Eating slowly lead to lower postprandial concentrations of the anorexigenic gut peptides peptide YY and glucagon-like peptide 1 (GLP-1) [14], [15]. These findings indicate that adequate eating habits prevent the incidence of diabetes by improving glucose metabolism after meals.

Recently, a few studies have reported an association between the rate of eating and the risk of diabetes [16], [17]. To date, however, the association between mastication, particularly masticatory performance, and diabetes has not been clarified.

Here, we investigated the association between mastication, namely masticatory performance or rate of eating, and diabetes in a population-based cohort.

## Methods

### Study design and population

We conducted a cross-sectional study of the association between masticatory performance and diabetes. Subjects were participants in the Nagahama Prospective Genome Cohort for Comprehensive Human Bioscience (the Nagahama Study) [18]. The Nagahama Study is a longitudinal genetic epidemiological study aimed at clarifying as-yet unidentified factors and pathways relating genetic variants and disease phenotypes of common diseases and disorders, such as cardiovascular diseases, endocrine and metabolic diseases, immunological diseases and oral diseases via the comprehensive analysis of omics data. The Nagahama Study participants were recruited from apparently healthy community residents aged 30 to 74 years living in Nagahama City, a largely rural city of approximately 125,000 inhabitants in Shiga Prefecture, located in the center of Japan. A total of 9,804 participants were recruited during 2008 to 2010. Among 8,679 participants who underwent oral examination and masticatory performance tests in 2009–2010, inclusion in the present study was restricted to participants aged 40 years or older who completed all baseline measurements, and participants who were pregnant or who had a history of gestational diabetes were excluded. After applying these eligibility criteria, a total of 6,827 participants were entered into the analysis.

### Ethics statement

This study protocol was approved by the Ethics Committee of Kyoto University Graduate School and Faculty of Medicine, the Ethical Review Board of the Nagahama Study, and the Nagahama Municipal Review Board of Personal Information Protection. Written informed consent was obtained from all participants.

### Measurement of masticatory performance

Participants were instructed to masticate a piece of chewing gum (Lotte Co., Ltd.) as usual for one minute. Participants who had a denture were instructed to wear it and masticate. The chewing gum changes color as mastication proceeds. After each trial, the chewed gum bolus was placed between two plastic films and pressed into an approximately 30-mm diameter disk. Color measurement using a CR-13 spectrophotometer (Konica Minolta Holdings, Inc.) was performed through the plastic films at five sites, one in the center and four others in the midpoint of imaginary spoke lines extending from the center to the superior, inferior, left, and right margins on the surface of the flattened gum [19].

Measurement was performed by an experienced dentist. Color was evaluated using the L*a*b* color space, which was developed by the Commission Internationale de l'Eclairage (CIE) for measuring object color [20]. Mean values of L*a*b* measured at the five sites on the gum were used to estimate the color difference ΔE*ab, which is calculated by the following equation: ΔE*ab  =  {(ΔL*)^ 2^ + (Δa*)^ 2^ + (Δb*)^ 2^}^1/2^ [20]. Respective values of L*a*b* before chewing were 73.1, −11.6, and 34.4 (means). We regarded the estimated ΔE*ab as the masticatory performance of the participant [21], [22]. We then divided participants into four groups by quartile of masticatory performance.

### Definition of diabetes

The value for HbA1c (%) was estimated as an National Glycohemoglobin Standardization Program (NGSP)-equivalent value (%), calculated by the formula A1C (%)  =  A1C (Japan Diabetes Society (JDS)) (%) + 0.4%, in consideration of the relational expression of HbA1c (JDS) (%) measured by the previous Japanese standard substance and measurement methods and A1C (NGSP) [23]. Participants were considered diabetic if they met at least one of the following parameters: fasting blood glucose level ≥126 mg/dl (≥7.0 mmol/l), random plasma glucose level ≥200 mg/dl (≥11.1 mmol/l), or HbA1c ≥6.5% (HbA1c ≥6.1% according to JDS) [23]. Diabetes was diagnosed if the blood sample was confirmed to be a diabetic type both by plasma glucose level and HbA1c at the same time [23], or the participant had received any treatment with hypoglycemic medication (hypoglycemic agent and/or insulin). Fasting was defined as no caloric intake for at least 8 hours [24].

### Dependent variables

The health examination included height, weight, blood pressure, and blood tests. Two sitting blood pressure measurements were averaged for analysis. Blood samples from each participant were used to measure total cholesterol, high-density cholesterol, triglyceride, plasma glucose, and HbA1c. In addition, oral examinations were conducted by two trained dentists, with an inter-examiner reliability of 0.77 to 1.0 by kappa statistics. Oral examination included DMF index and periodontal status, excluding third molars. The DMF index, which comprises the number of decayed (D), missing (M), and filled (F) teeth, has been established as a key measurement of caries experience in dental epidemiology [25]. We then estimated the number of present teeth as 28 – the number of missing teeth. Periodontal status was assessed according to the Community Periodontal Index (CPI) [25]. CPI was used to assess the presence or absence of periodontal disease in each sextant (i.e. sixth of dentition) according to the following items: (Score 0) healthy, (Score 1) gingival bleeding after probing, (Score 2) supra- or sub-gingival calculus, (Score 3) pocket depth between 4 and 5 mm and, (Score 4) pocket depth between 6 mm or more. We used a standardized lightweight periodontal probe with a 0.5-mm ball tip to probe standardized index teeth and categorized periodontal status with a score of 0 to 4. Index teeth were investigated as the recommended 10 teeth; if the index tooth was missing, the next adjacent tooth was used for evaluation [26]. Subjects with complete edentulousness were entered into a separate category for the calculation of CPI [26].

A self-administered questionnaire was used to assess medical history, including history of diabetes, type of hypoglycemic medication, and history of diabetes in a first-degree relative (no or yes). In addition, life-style variables were surveyed, including smoking habit (never, former, or current), alcohol drinking habit (never, former, or current), physical activity (slight, moderate, or strenuous), caloric restriction (no, yes at subject discretion, or yes due to medical diagnosis) and rate of eating (slow, intermediate, or fast). We also surveyed the intake of 18 kinds of foods, including rice, meat, fish, tofu (soybean curd), eggs, milk, and vegetables using a self-answered questionnaire, with categories of every day, 4–5 times per week, 2–3 times per week, and l time or less per week.

### Statistical analysis

All analyses were stratified by sex. A nonparametric test for trend across ordered groups were performed. Spearman correlation coefficients were used to assess the association between masticatory performance and the other continuous variables. To adjust for demographic and possible confounding factors, logistic regression analysis was performed with diabetes as a dependent variable, and the odds ratio (OR) of diabetes and 95% confidence interval (CI) were estimated in three models: Model 1, crude; Model 2, adjusted for age (40–49, 50–64, 65–74); and Model 3, adjusted for model 2 and other possible confounding factors. In the selection of variables, we used a forced entry method and entered the following variables into the model as possible confounding factors: age, body mass index (BMI) (<18.5, 18.5–24.9, 25.0–29.9 or ≥30.0), family history of diabetes (no or yes), current smoking (no or yes), current alcohol drinking (no or yes), physical activity (slight, moderate or strenuous), caloric restriction (no, yes at subject discretion, or yes due to medical diagnosis), rate of eating (slow, intermediate, or fast) and periodontal status (no pathological pockets (score 0, 1 and 2), periodontal pockets (score 3 and 4), or edentulous). Goodness of fit of the model was examined using the Hosmer-Lemeshow goodness of fit test. Linear tests for trend were performed with the median value in each group as a continuous variable [27]. All *P* values were two sided at a significance level of 5%. All statistical analyses were performed using Stata 11.2 software (Stata Corporation, College Station, TX, USA).

## Results

The distribution of masticatory performance and prevalence of diabetes in the participants are shown in Appendix S1. Masticatory performance was lower and the prevalence of diabetes was higher with age in both males and females. Large differences in the prevalence of diabetes between males and females were seen for all age groups, with an average prevalence of 7.7% in males, 2.4% in females, and 4.2% in all.

Masticatory performance was divided into quartiles, namely quartiles one (1.85 to 34.21), two (34.22 to 40.45), three (40.47 to 46.12) and four (46.14 to 59.9) in males, and one (2.63 to 32.44), two (32.45 to 38.21), three (38.22 to 43.05) and four (43.06 to 56.15) in females. We divided participants into four groups according to quartile of masticatory performance, namely Q1 (lowest), 2, and 3 and 4 (highest) groups. Characteristics of males and females by masticatory performance are shown in Tables 1 and 2. A positive correlation between masticatory performance and height was seen in both males and females. Similar correlations were seen in weight and BMI in males, but not in females. Regarding lifestyle, the rate of smokers (former and current) was lower as masticatory performance increased in both males and females. In addition, the rate of participants eating fast was higher and the prevalence of periodontitis was lower. In contrast, no large differences were found among the four groups regardless of sex in blood pressure, family history of diabetes, physical activity, or caloric restriction.

**Table 1:** Characteristics of male participants by masticatory performance.

	Masticatory performance				
	Q1 (lowest)	Q2	Q3	Q4 (highest)	*P* trend
Participants, *n*	572	570	571	570	
Age, *y*	65.0 (59.0–69.0)	63.0 (56.0–68.0)	62.0 (54.0–67.0)	61.0 (54.0–67.0)	<0.001
Height, *cm*	166.6 (162.4–170.9)	167.1 (163.0–171.2)	167.2 (163.3–171.7)	167.9 (163.7–172.1)	<0.001
Weight, *kg*	64.1 (57.8–71.0)	64.7 (58.7–70.7)	65.6 (59.6–71.6)	65.2 (60.3–72.7)	<0.001
Body mass index, *kg/m^2^*	23.1 (21.2–25.0)	23.2 (21.1–25.1)	23.3 (21.7–25.1)	23.5 (21.5–25.2)	0.018
Blood pressure, *mm Hg*					
Systolic	128.5 (120.0–141.0)	127.0 (118.0–138.0)	128.0 (119.0–139.0)	129.0 (119.0–140.0)	0.94
Diastolic	80.0 (73.0–87.0)	80.0 (74.0–87.0)	80.0 (73.0–88.0)	82.0 (75.0–89.0)	0.004
HbA1c, %	5.6 (5.3–5.9)	5.5 (5.3–5.8)	5.5 (5.3–5.8)	5.5 (5.3–5.8)	0.075
Glucose level, *mg/dl*	92.0 (86.0–101.0)	91.0 (85.0–98.0)	91.0 (85.0–97.0)	91.0 (86.0–98.0)	0.097
Total cholesterol, *mg/dl*	200.0 (177.0–221.0)	197.5 (179.0–222.0)	203.0 (182.0–222.0)	203.0 (182.0–229.0)	0.003
High-density cholesterol, *mg/dl*	54.0 (45.0–65.0)	55.0 (45.0–67.0)	56.0 (47.0–69.0)	57.0 (48.0–67.0)	0.002
Triglyceride, *mg/dl*	105.0 (74.0–149.5)	100.5 (70.0–140.0)	102.0 (74.0–146.0)	101.0 (72.0–143.0)	0.53
Prevalence of diabetes, *n* (%)	57 (9.9)	48 (8.4)	42 (7.3)	30 (5.2)	0.002
Family history of diabetes, *n* (%)					
No	466 (81.4)	441 (77.3)	450 (78.8)	460 (80.7)	0.90
Yes	106 (18.5)	129 (22.6)	121 (21.1)	110 (19.3)	
Smoking, *n* (%)					
Never	90 (15.7)	128 (22.4)	144 (25.2)	170 (29.8)	<0.001
Former	258 (45.1)	273 (47.8)	310 (54.2)	283 (49.6)	
Current	224 (39.1)	169 (29.6)	117 (20.4)	117 (20.5)	
Alcohol drinking, *n* (%)					
Never	97 (16.9)	75 (13.1)	73 (12.7)	58 (10.1)	0.001
Former	21 (3.6)	19 (3.3)	16 (2.8)	17 (2.9)	
Current	454 (79.3)	476 (83.5)	482 (84.4)	495 (86.8)	
Physical activity, *n* (%)					
Slight	61 (10.6)	75 (13.1)	56 (9.8)	56 (9.8)	0.86
Moderate	344 (60.1)	347 (60.8)	349 (61.1)	359 (62.9)	
Strenuous	167 (29.2)	148 (25.9)	166 (29.0)	155 (27.1)	
Caloric restriction, *n* (%)					
No	378 (66.0)	378 (66.3)	382 (66.9)	398 (69.8)	0.28
Yes, at subject discretion	168 (29.3)	164 (28.7)	153 (26.8)	139 (24.3)	
Yes, due to medical diagnosis	26 (4.5)	28 (4.9)	36 (6.3)	33 (5.7)	
Rate of eating, *n* (%)					
Slow	61 (10.6)	55 (9.6)	52 (9.1)	37 (6.4)	0.006
Intermediate	313 (54.7)	302 (52.9)	295 (51.6)	301 (52.8)	
Fast	198 (34.6)	213 (37.3)	224 (39.2)	232 (40.7)	
Periodontal status, *n* (%)					
Healthy	75 (13.1)	54 (9.4)	80 (14.0)	64 (11.2)	<0.001
Gingival bleeding	16 (2.8)	35 (6.1)	56 (9.8)	46 (8.0)	
Supra- or sub-gingival calculus	59 (10.3)	74 (12.9)	81 (14.1)	113 (19.8)	
Shallow periodontal pockets	145 (25.3)	177 (31.0)	169 (29.6)	169 (29.6)	
Deep periodontal pockets	220 (38.4)	216 (37.8)	180 (31.5)	176 (30.8)	
Edentulous	57 (9.9)	14 (2.4)	5 (0.8)	2 (0.3)	

Continuous variables are presented as medians (interquartile range).

Categorical variables are presented as numbers (%).

**Table 2:** Characteristics of female participants by masticatory performance.

	Masticatory performance				
	Q1 (lowest)	Q2	Q3	Q4 (highest)	*P* trend
Participants, *n*	1,139	1,136	1,133	1,136	
Age, *y*	61.0 (54.0–67.0)	59.0 (50.5–65.0)	59.0 (50.0–64.0)	59.0 (52.0–65.0)	<0.001
Height, *cm*	154.3 (150.4–158.2)	155.2 (151.1–159.1)	155.5 (151.7–159.2)	155.1 (151.1–159.1)	<0.001
Weight, *kg*	52.6 (47.4–57.9)	52.1 (48.1–57.4)	52.1 (47.7–57.4)	52.5 (48.1–57.6)	0.58
Body mass index, *kg/m^2^*	22.0 (20.2–24.2)	21.7 (19.9–23.8)	21.4 (19.8–23.7)	21.9 (20.1–23.9)	0.18
Blood pressure, *mm Hg*					
Systolic	123.0 (112.0–134.0)	120.0 (109.0–131.0)	121.0 (110.0–132.0)	122.0 (111.0–133.0)	0.15
Diastolic	76.0 (68.0–83.0)	74.0 (67.0–81.0)	74.0 (68.0–82.0)	75.0 (68.0–82.0)	0.83
HbA1c, %	5.5 (5.3–5.7)	5.5 (5.3–5.7)	5.5 (5.3–5.7)	5.5 (5.3–5.7)	0.12
Glucose level, *mg/dl*	89.0 (84.0–93.0)	88.0 (83.0–93.0)	88.0 (84.0–93.0)	88.0 (84.0–93.0)	0.58
Total cholesterol, *mg/dl*	215.0 (193.0–237.0)	213.0 (192.0–234.0)	215.0 (192.0–238.0)	216.0 (197.0–239.0)	0.036
High-density cholesterol, *mg/dl*	65.0 (55.0–77.0)	67.0 (56.0–78.0)	68.0 (57.0–80.0)	69.0 (57.0–81.0)	<0.001
Triglyceride, *mg/dl*	84.0 (61.0–118.0)	78.0 (60.0–112.5)	77.0 (57.0–108.0)	79.0 (59.0–109.5)	0.001
Prevalence of diabetes, *n* (%)	33 (2.9)	34 (2.9)	28 (2.4)	17 (1.5)	0.022
Family history of diabetes, *n* (%)					
No	886 (77.7)	860 (75.7)	852 (75.2)	859 (75.6)	0.21
Yes	253 (22.2)	276 (24.3)	281 (24.8)	277 (24.3)	
Smoking, *n* (%)					
Never	1,002 (87.9)	1,024 (90.1)	1,029 (90.8)	1,044 (91.9)	0.001
Former	57 (5.0)	68 (5.9)	69 (6.0)	59 (5.1)	
Current	80 (7.0)	44 (3.8)	35 (3.0)	33 (2.9)	
Alcohol drinking, *n* (%)					
Never	619 (54.3)	554 (48.7)	545 (48.1)	565 (49.7)	0.015
Former	21 (1.8)	6 (0.5)	11 (0.9)	7 (0.6)	
Current	499 (43.8)	576 (50.7)	577 (50.9)	564 (49.6)	
Physical activity, *n* (%)					
Slight	64 (5.6)	84 (7.3)	67 (5.9)	68 (5.9)	0.76
Moderate	856 (75.1)	816 (71.8)	842 (74.3)	840 (73.9)	
Strenuous	219 (19.2)	236 (20.7)	224 (19.7)	228 (20.0)	
Caloric restriction, *n* (%)					
No	633 (55.5)	636 (55.9)	575 (50.7)	601 (52.9)	0.090
Yes, at subject discretion	451 (39.6)	455 (40.0)	500 (44.1)	495 (43.5)	
Yes, due to medical diagnosis	55 (4.8)	45 (3.9)	58 (5.1)	40 (3.5)	
Rate of eating, *n* (%)					
Slow	125 (10.9)	89 (7.8)	120 (10.5)	84 (7.3)	0.025
Intermediate	678 (59.5)	684 (60.2)	652 (57.5)	677 (59.6)	
Fast	336 (29.5)	363 (31.9)	361 (31.8)	375 (33.0)	
Periodontal status, *n* (%)					
Healthy	230 (20.1)	197 (17.3)	206 (18.1)	192 (16.9)	<0.001
Gingival bleeding	110 (9.6)	162 (14.2)	148 (13.0)	141 (12.4)	
Supra- or sub-gingival calculus	150 (13.1)	171 (15.0)	217 (19.1)	248 (21.8)	
Shallow periodontal pockets	333 (29.2)	378 (33.2)	352 (31.0)	351 (30.9)	
Deep periodontal pockets	276 (24.2)	220 (19.3)	207 (18.2)	203 (17.8)	
Edentulous	40 (3.5)	8 (0.7)	3 (0.2)	1 (0.1)	

Continuous variables are presented as medians (interquartile range).

Categorical variables are presented as numbers (%).

We investigated the association between masticatory performance and prevalence of diabetes and found an inverse dose-dependent association (Table 3). Compared to the lowest group as a reference, crude odds ratio of diabetes was 0.83 (95% CI, 0.55–1.2) in Q2, 0.71 (95% CI, 0.47–1.0) in Q3 and 0.50 (95% CI, 0.31–0.79) in the high group in males, and 1.0 (95% CI, 0.63–1.6) in Q2, 0.84 (95% CI, 0.50–1.4) in Q3 and 0.50 (95% CI, 0.28–0.91) in the high group in females. These trends were still observed in males after adjustment for age (*P* for trend  = 0.015) and for demographic and possible confounding factors (*P* for trend  = 0.031). The multivariable adjusted OR of diabetes was 0.91 (95% CI, 0.58–1.4) in Q2, 0.77 (95% CI, 0.48–1.2) in Q3 and 0.53 (95% CI, 0.31–0.90) in the high group. In contrast, we found no significant association between masticatory performance and diabetes in females. The final multivariable adjusted model was reliable (*P* = 0.62 in males and *P* = 0.70 in females by the Hosmer–Lemeshow test).

**Table 3:** Masticatory performance and risk of diabetes.

	Masticatory performance				
	Q1 (lowest)	Q2	Q3	Q4 (highest)	*P* trend
Male					
Number of subjects with diabetes	57	48	42	30	
Number of subjects without diabetes	515	522	529	540	
Crude (95% CI)	1.0 (ref)	0.83 (0.55–1.2)	0.71 (0.47–1.0)	0.50 (0.31–0.79)	0.003
Age-adjusted OR (95% CI)	1.0 (ref)	0.89 (0.59–1.3)	0.77 (0.50–1.1)	0.55 (0.34–0.87)	0.015
Multivariable-adjusted OR (95% CI)	1.0 (ref)	0.91 (0.58–1.4)	0.77 (0.48–1.2)	0.53 (0.31–0.90)	0.031
Female					
Number of subjects with diabetes	33	34	28	17	
Number of subjects without diabetes	1,106	1,102	1,105	1,119	
Crude (95% CI)	1.0 (ref)	1,0 (0.63–1.6)	0.84 (0.50–1.4)	0.50 (0.28–0.91)	0.028
Age-adjusted OR (95% CI)	1.0 (ref)	1.1 (0.70–1.8)	0.96 (0.57–1.6)	0.55 (0.30–0.99)	0.074
Multivariable-adjusted OR (95% CI)	1.0 (ref)	1.2 (0.73–2.0)	0.95 (0.54–1.6)	0.56 (0.30–1.0)	0.083

OR  =  odds ratio; CI  =  confidence interval; ref  =  reference.

Multivariate ORs for diabetes were adjusted for age (40–49, 50–64 or 65–74), body mass index (<18.5, 18.5–24.9, 25.0–29.9 or ≥30.0), family history of diabetes (no or yes), current smoking (no or yes), current alcohol drinking (no or yes), physical activity (slight, moderate, or strenuous), caloric restriction (no, yes at subject discretion, or yes due to medical diagnosis), rate of eating (slow, intermediate, or fast) and periodontal status (no pathological pockets (score 0, 1 and 2), periodontal pockets (score 3 and 4), or edentulous).

We also conducted an additional analysis and estimated OR of diabetes by the rate of eating (Table 4). As with masticatory performance, we found that slow eating was significantly associated with decreased odds for diabetes in multivariable adjusted ORs in males (*P* for trend  = 0.048). In contrast, we found no significant association in females.

**Table 4:** Rate of eating and risk of diabetes.

	Rate of eating			
	Fast	Intermediate	Slow	*P* trend
Male				
Number of subjects with diabetes	76	94	7	
Number of subjects without diabetes	791	1,117	198	
Crude (95% CI)	1.0 (ref)	0.87 (0.63–1.2)	0.36 (0.16–0.81)	0.026
Age-adjusted OR (95% CI)	1.0 (ref)	0.77 (0.56–1.0)	0.30 (0.13–0.67)	0.002
Multivariable-adjusted OR (95% CI)	1.0 (ref)	0.87 (0.61–1.2)	0.38 (0.16–0.91)	0.048
Female				
Number of subjects with diabetes	39	61	12	
Number of subjects without diabetes	1,396	2,630	406	
Crude (95% CI)	1.0 (ref)	0.83 (0.55–1.2)	1.0 (0.54–2.0)	0.75
Age-adjusted OR (95% CI)	1.0 (ref)	0.79 (0.52–1.1)	0.96 (0.49–1.8)	0.50
Multivariable-adjusted OR (95% CI)	1.0 (ref)	0.92 (0.59–1.4)	1.5 (0.73–3.0)	0.65

OR  =  odds ratio; CI  =  confidence interval; ref  =  reference.

Multivariate ORs for diabetes were adjusted for age (40–49, 50–64, or 65–74), body mass index (<18.5, 18.5–24.9, 25.0–29.9, or ≥30.0), family history of diabetes (no or yes), current smoking (no or yes), current alcohol drinking (no or yes), physical activity (slight, moderate, or strenuous), caloric restriction (no, yes at subject discretion, or yes due to medical diagnosis), masticatory performance (Q1, Q2, Q3 or Q4) and periodontal status (no pathological pockets (score 0, 1 and 2), periodontal pockets (score 3 and 4), or edentulous).

## Discussion

We examined the association between mastication and diabetes in a population-based cohort. An inverse dose-dependent association was observed between masticatory performance and diabetes in both males and females in the estimation of crude odds ratio. The association was maintained in males after adjustment for potential confounding factors. In addition, slow eating was significantly associated with decreased odds for diabetes in males. In females, in contrast, no associations were found after adjustment, albeit that this might have been due to the low prevalence of diabetes in females in our study population. To our knowledge, this is the first study to clarify the association between mastication, namely masticatory performance or rate of eating, and diabetes.

We hypothesize two possible mechanisms underlying the association between masticatory performance and diabetes. The first involves the reduced intake of nutrients such as dietary fiber or magnesium, which were lower in subjects who were unable to fully masticate due to teeth loss, ill-fitting dentures or edentulousness [10]–[12]. Indeed, insufficient dietary fiber, magnesium or calcium intakes were reported to be associated with the risk of type 2 diabetes [3], [4], [28]–[31]. In particular, the intake of dietary fiber reduces glucose and influences insulin responses as a result of the retarding effect of soluble fiber on gastric emptying and absorption [32]. The other mechanism underlying the association involves the habitual chewing of hard food. A hard gum chewing exercise was effective in increasing maximum bite force and masticatory performance, and the effects were maintained after exercise completion [33]. Habitual chewing of hard foods was also reported to influence body weight loss, postprandial thermogenesis and glucose metabolism, although the mechanism of these effects remains unclear [34], [35]. In addition, hardness of the habitual diet was an important environmental factor in the prevention of diabetes in a mouse model [36]. These previous studies strongly support our present findings.

Second, we found that slow eating was significantly associated with decreased odds for diabetes in males, after considering dental problems and other potential confounding factors. Recent studies have found that eating fast by self-assessed questionnaire was associated with a higher risk of diabetes in middle-aged Japanese males [16], and an increased HbA1c level in diabetic patients treated with insulin [17]. In particular, Sakurai et al. reported multivariate-adjusted hazard ratios (95% CI) of 1.00 (reference) among the slow group, versus 1.68 (0.93–3.02) among the intermediate group and 1.97 (1.10–3.55) among the fast group in a 7-year cohort [16]. These results appear similar to ours; however, their target population consisted of employee or hospital-registered patients whereas ours consisted of community residents, and oral status in their population was not evaluated. A second interesting observation was that fast eating was independently and positively associated with insulin resistance [37].

The mechanism underlying the association between rate of eating and glucose metabolism may be elucidated from the following studies. Lengthening mastication (thoroughness of mastication) was reported to elicit lower postprandial plasma glucose concentrations, because of the potentiation of early-phase insulin secretion [13]. Eating slowly also increased the postprandial response of the anorexigenic gut peptides GLP-1, which plays an important role in enhancing the glucose-stimulated insulin secretion of β-cells, and peptide YY, which regulates hunger, satiety, and energy intake [14], [15]. Moreover, several studies have shown an association between fast eating and higher BMI or weight gain [5]–[7], [17], [38]. A possible explanation of these findings is that overeating or increased energy intake in fast eaters was a result of a defect in hypothalamic neural histamine [5], [39], or a lowering of satiety signals transmitted to the brain, which are triggered on nutrient ingestion by gastric distension and the release of gut factors, including cholecystokinin [40]. These reports may support the argument that eating food slowly – masticating food well – prevents obesity or insulin resistance diabetes.

Mastication or chewing serves several functions, namely the breakdown of large food particles into smaller particles suitable for gastrointestinal absorption of nutrients; and lubricating and softening food particles into a bolus conducive to swallowing, thereby facilitating gastrointestinal absorption of food particles [41]. The quality of mastication can be evaluated as masticatory performance, which is determined as the capacity to reduce the size of food particles, for example almonds, by chewing for a standardized period of time. Masticatory performance has also been determined as the number of chews necessary to render food ready for swallowing [41]. Masticatory performance in the present study was evaluated using a color-changeable chewing gum, as used in a number of other studies [19], [21], [22], [33], [42]–[46]. Participants were instructed to chew the gum as usual regardless of the number of chews, because the focus of our study was to evaluate their regular ability to masticate food in unit time. In addition, the method is simple and quantitative, and its validity and reliability have been confirmed [44]–[46], with correlation coefficients for intra- and inter-examiner consistency of more than 0.88 for three different groups (dentists, adults and elderly people) [44], and significant correlation coefficient between masticatory performance and the scores of patient satisfaction questionnaires or food questionnaires [45] or number of chewable foods [46]. Hayakawa et al. quantified the chromaticity coordinate “a” only, which represents the degree of red color, on the basis that the gum color changes from purple-blue to red as mastication proceeds [19]. In 1976, however, CIE proposed the use of ΔE*ab for small color differences in the L*a*b* color space and for differences which result from colorant mixtures [20], and this has been supported by many researchers [21], [22], [47]. In addition, some participants in this population could not masticate fully and their chewed gum bolus contained colorant mixtures with red and green. We therefore decided to use ΔE*ab to evaluate masticatory performance.

Participants who answered that they ate slowly were more common in the lower masticatory performance groups. In the multivariate analysis, in contrast, slow eating was associated with decreased odds for diabetes. This result appears contradictory. Although the speed of chewing, namely the number of chewing strokes per minute, was reported to be related with masticatory performance [21], [43], [44], [48], the association between categorical rate of eating and masticatory performance or speed of chewing is unclear. Further investigation of the association between categorical rate of eating and masticatory performance or speed of chewing in a different population is required.

Several limitations of this study warrant mention. First, it was a cross-sectional study, and a follow-up survey is accordingly required to draw causal conclusions. For instance, diabetes may influence masticatory performance by increasing susceptibility to infections and thus the risk of periodontal disease [49], which in turn decreases masticatory performance due to teeth loss [50]. Second, total calorie intake was not investigated in this study and “caloric restriction” from a self-reported questionnaire was used as a surrogate. Instead, we surveyed the intake of 18 kinds of foods. Results showed no large differences in their distribution among the four groups by quartile of masticatory performance (data not shown). Third, we were unable to examine in detail dental prosthesis condition or type of dentition, which may be associated with masticatory performance [45], [46], [50]. In addition, a number of physical characteristics of participants which are involved in mastication were unclear, namely the action of the teeth, masticatory muscles, temporomandibular joint, tongue and saliva [41], [51]. This lack of examination might have resulted in underestimation of the extent of masticatory performance. Fourth, socioeconomic variables such as education or income were not collected in this cohort.

In conclusion, we identified an inverse dose-dependent association between masticatory performance and diabetes in a population-based cohort. After adjustment for possible confounding factors, odds of diabetes decreased gradually as masticatory performance increased. In addition, fast eating was found to be a possible risk factor for the development of diabetes. Taken together, the present and previous results indicate that slow eating and preservation of high masticatory performance by the prevention of tooth loss or maintenance of dental prosthesis might prevent the occurrence of diabetes. These are potentially modifiable factors, and this study provides important new information for physicians and dentists concerned with the prevention of diabetes.

## Supporting Information

Appendix S1 Distribution of masticatory performance and prevalence of diabetes stratified by sex and age in the Nagahama cohort.
(DOCX)
