# Supplementary material for: Mastication and Risk for Diabetes in a Japanese Population: A Cross-Sectional Study
Source: PLoS One. 2013 Jun 5;8(6):e64113. doi: 10.1371/journal.pone.0064113 (PMC3674007; doi:10.1371/journal.pone.0064113)
Supplement: Appendix S1 — Distribution of masticatory performance and prevalence of diabetes stratified by sex and age in the Nagahama cohort. (DOCX) [file pone.0064113.s001.docx]

Appendix S2. Distribution of masticatory performance and prevalence of diabetes stratified by sex and age in the Nagahama cohort

|  |  | Masticatory performance |  | Prevalence of diabetes |
| --- | --- | --- | --- | --- |
|  | number | median (IQR) |  | number (%) |
| Male |  |  |  |  |
| 40-49 | 346 | 41.9 (36.4-47.2) |  | 4 (1.1) |
| 50-59 | 438 | 41.7 (35.1-46.8) |  | 25 (5.7) |
| 60-69 | 1,079 | 39.9 (33.8-45.8) |  | 107 (9.9) |
| 70-74 | 420 | 38.9 (32.5-44.7) |  | 41 (9.7) |
| Total | 2,283 | 40.4 (34.2-46.1) |  | 177 (7.7) |
| Female |  |  |  |  |
| 40-49 | 922 | 38.5 (33.5-42.8) |  | 6 (0.6) |
| 50-59 | 1,349 | 38.8 (33.6-43.7) |  | 17 (1.2) |
| 60-69 | 1,742 | 38.0 (31.8-42.9) |  | 69 (3.9) |
| 70-74 | 531 | 35.7 (28.4-41.4) |  | 20 (3.7) |
| Total | 4,544 | 38.2 (32.4-43.0) |  | 112 (2.4) |

IQR = interquartile range.
